# Mitochondria Donation by Mesenchymal Stem Cells: Current Understanding and Mitochondria Transplantation Strategies

**DOI:** 10.3389/fcell.2021.653322

**Published:** 2021-04-07

**Authors:** Marina O. Gomzikova, Victoria James, Albert A. Rizvanov

**Affiliations:** ^1^Institute of Fundamental Medicine and Biology, Kazan Federal University, Kazan, Russia; ^2^M.M. Shemyakin–Yu.A. Ovchinnikov Institute of Bioorganic Chemistry of the Russian Academy of Sciences, Moscow, Russia; ^3^School of Veterinary Medicine and Science, University of Nottingham, Nottingham, United Kingdom

**Keywords:** mitochondria donation, mitochondria transplantation, tunneling nanotubes, extracellular vesicles, cell fusion, isolated mitochondria

## Abstract

The phenomenon of mitochondria donation is found in various tissues of humans and animals and is attracting increasing attention. To date, numerous studies have described the transfer of mitochondria from stem cells to injured cells, leading to increased ATP production, restoration of mitochondria function, and rescue of recipient cells from apoptosis. Mitochondria transplantation is considered as a novel therapeutic approach for the treatment of mitochondrial diseases and mitochondrial function deficiency. Mitochondrial dysfunction affects cells with high energy needs such as neural, skeletal muscle, heart, and liver cells and plays a crucial role in type 2 diabetes, as well as Parkinson’s, Alzheimer’s diseases, ischemia, stroke, cancer, and age-related disorders. In this review, we summarize recent findings in the field of mitochondria donation and mechanism of mitochondria transfer between cells. We review the existing clinical trials and discuss advantages and disadvantages of mitochondrial transplantation strategies based on the injection of stem cells, isolated functional mitochondria, or EVs containing mitochondria.

## Introduction

Mitochondria are key players in the cell’s energy production, calcium homeostasis, signaling, and apoptosis (Rossi et al., 2019). Deficiency in mitochondrial function is observed in inherited mitochondrial diseases as well as cancer, diabetes, neurodegenerative diseases (including Alzheimer’s disease, Parkinson’s disease, and Huntington’s disease), aging, and age-related metabolic disorders—so-called mitochondria-associated diseases (Bhatti et al., 2017). Replacement of non-functional mitochondria by transplantation of healthy mitochondria into injured cells is believed to potentially be a universal solution for the treatment of mitochondrial deficiency of different etiologies. Delivery of even a few healthy mitochondria can lead to the sustained restoration of mitochondria function in a recipient cell.

Detection of mitochondria donation from mesenchymal stromal cells also known as mesenchymal stem cells (MSCs) to recipient cells aroused great interest in the field of regenerative medicine. Mitochondrial donation leads to the rescue of injured cells, improved oxidative phosphorylation, increased ATP production, and restoration of mitochondrial function (Rustom, 2016). As a result of these findings, it is now generally accepted that the reparative function of MSCs is partly mediated by mitochondrial transfer, which seems to be an evolutionarily conserved phenomenon. Horizontal transfer of mitochondria between mammalian cells provides a novel therapeutic approach for the treatment of mitochondrial and mitochondria-associated diseases and stimulates development of mitochondria transplantation strategies.

The development of a robust mitochondria delivery protocol is a critical issue that needs to be addressed prior to any widespread clinical application of mitochondrial transplantation. Due to the immunogenic complications of using isolated mitochondria (Ishikawa et al., 2010; Zhang et al., 2010; Wang et al., 2018), MSC-based mitochondria transfer is considered as a promising therapeutic strategy. Since it was first described by Islam et al. (2012) discovered that BMSCs transfer mitochondria to injured lung alveolar epithelial cells. MSC-based mitochondria transfer has been used for the treatment of diverse mitochondria-associated disorders such as ischemic stroke (Liu et al., 2019), spinal cord injury (Li et al., 2019), kidney injury (Zou et al., 2018), cardiomyoblast ischemia model (Cselenyak et al., 2010), and respiratory system injury (Islam et al., 2012). A more recent shift in the field of regenerative medicine, from cell based to cell-free therapy (Gomzikova and Rizvanov, 2017), has led to the study of new approaches, such as mitochondria coating with biocompatible polymers and encapsulation into microvesicles.

In this review, we provide an overview of recent findings in the field of mitochondria donation and mechanisms of mitochondria transfer. We discuss a therapeutic strategy based on injection of isolated functional mitochondria and describe advances and challenges of mitochondrial transplantation strategies, based on injection of stem cells, isolated functional mitochondria, or EVs containing mitochondria.

## MSCs Donate Mitochondria to Target Cells

The phenomenon of mitochondria transfer was first observed between endothelial progenitor cells and cardiac myocytes (Koyanagi et al., 2005). Subsequently, mitochondrial donation from MSCs was described by Spees et al. (2006). The authors demonstrated that after cocultivation of hMSCs with A549 ρ°cells (containing defective mtDNA), some A549 ρ°cells acquired functional mitochondria derived from donor hMSCs. Cells containing donor mitochondria showed active proliferation, decreased levels of reactive oxygen species, increased ATP production, membrane potential, and oxygen consumption (Spees et al., 2006). Furthermore, Plotnikov et al. (2008) also reported mitochondrial transfer when investigating the cocultivation of MSCs with rat cardiomyocytes or rat renal tubular cells (Plotnikov et al., 2010); the number of tunneling nanotubes (TNTs) significantly rose in correlation with the detection of mitochondrial transfer. Further studies have since confirmed the process of intercellular mitochondrial transfer *in vitro* (Acquistapace et al., 2011; Pankotai et al., 2012). With Islam et al. (2012) providing the first evidence of mitochondrial transfer *in vivo* (Islam et al., 2012), by demonstrating in an LPS-induced acute lung injury model, bone marrow-derived MSCs transfer mitochondria to the injured alveolar epithelial cells inducing generation of ATP and increasing mouse survival (Islam et al., 2012). In addition to mitochondrial donation from MSCs, mitochondrial donation has also been observed from endothelial cells to cancer cells (Pasquier et al., 2013), from astrocytes to neurons (Hayakawa et al., 2016), and from cancer-associated fibroblasts to prostate cancer cells (Ippolito et al., 2019).

Since these first studies reported the existence of intercellular mitochondria transfer, numerous studies have gone on to demonstrate that MSCs donate mitochondria leading to the rescue of the injured cell, improved aerobic respiration, and inhibited apoptosis. This has been demonstrated to occur in endothelial cells within *in vitro* ischemia–reperfusion models (Liu et al., 2014), as well as observations of attenuation of alveolar destruction and altered severity of fibrosis in models of cigarette smoke-induced damage (Li et al., 2014), neuroprotective effects and decline of infarct volume in the brain (Babenko et al., 2015), amelioration of acute renal ischemia reperfusion injury (Gu et al., 2016), recovery of mitochondrial function in rat cardiomyocytes *in vitro* after ischemia/reperfusion injury (Han et al., 2016), protection of corneal epithelial cells from Rotenone-induced oxidative damage (Jiang et al., 2016), and decreased mutation ratio and oxidative damage in cells derived from a patient with mitochondrial disease (MERRF syndrome) (Chuang et al., 2017).

MSCs are readily attracted to tumor stroma. Studies of the tumor microenvironment have also demonstrated that MSCs can donate mitochondria to cancer cells, inducing their chemoresistance (Pasquier et al., 2013; Moschoi et al., 2016) and restoring impaired mitochondria function (Lin et al., 2015). Transfer of normal mitochondria from human umbilical cord-derived MSCs into breast cancer MDA-MB-231 cells increased the proliferation and invasiveness of MDA-MB-231 cells as well as enhance cisplatin-induced apoptosis (Kheirandish-Rostami et al., 2020). However, in contrast to MSCs, mitochondria isolated from normal human astrocytes inhibited malignant proliferation of human glioma U87 cells, as well as increasing aerobic respiration, attenuating glycolysis, and enhancing radiosensitivity both *in vitro* and *in vivo* (Sun et al., 2019). This phenomenon underlines the complexity and significance of the tumor microenvironment in cancer progression. Future research of the mechanisms of mitochondrial transfer between tumor and tumor-associated cells will hopefully provide new insights into potential therapeutic targets.

MSC mitochondrial transfer has also been observed to regulate immune cell activity. MSCs can deliver mitochondria to activated T cells, improving their energy state and suppressing aberrant autophagy in systemic lupus erythematosus (SLE) patients (Chen et al., 2016). The authors suggested that regulation of the energy state of T cells by mitochondrial transfer could be a new therapeutic strategy in SLE treatment. Mitochondrial transfer from MSC to macrophages results in enhanced alveolar macrophages with increases in phagocytosis, oxidative phosphorylation, and antimicrobial effects *in vivo* in the context of acute respiratory distress syndrome (ARDS) (Jackson et al., 2016; Jackson and Krasnodembskaya, 2017). It is believed that mitochondrial donation is a novel mechanism of MSC-mediated antimicrobial effects, mediated by enhancement of macrophage phagocytic activity. Morrison et al. (2017) revealed a novel mechanism of modulation of macrophage polarization through mitochondrial donation. The authors showed that MSCs transfer functional mitochondria enclosed in EVs, inducing M2 macrophage polarization and enhancement of phagocytic capacity, protecting against endotoxin-induced lung injury and ameliorating lung injury *in vivo* (Morrison et al., 2017). Regulation of T cell function by mitochondrial transfer was observed under cocultivation of T helper 17 (Th17) cells with bone marrow-derived MSCs (Luz-Crawford et al., 2019). The authors showed that pro-inflammatory Th17 cells acquire an anti-inflammatory phenotype after the mitochondria were transferred, whereas a reduction of transferred mitochondria may contribute to the chronic inflammation seen in rheumatoid arthritis (RA) synovitis (Luz-Crawford et al., 2019). The concept of organelle−based therapy for the treatment of immune diseases was demonstrated using a graft versus host disease (GvHD) mouse model (Court et al., 2020). Transplantation of human T cells treated with mitochondria led to a significant improvement in survival and reduction in tissue damage (Court et al., 2020).

In recent years, the number of articles describing mitochondria transfer has increased tremendously. We have summarized the body of research in which mitochondrial transfer between stem cells and recipient cells was detected, as well as mitochondrial transplantation in various disease models in Supplementary Table 1.

Injury and stress signals were shown to trigger the transfer of mitochondria from MSCs to recipient cells. Mitochondrial donation by MSCs was observed in mtDNA-deficient cells and mitochondrial toxin-treated cells, whereas mitochondrial transfer was not detected in cells harboring pathogenic mutations (Cho et al., 2012). The process of mitochondrial donation by MSCs was triggered by damaged somatic cell-derived mitochondria (Mahrouf-Yorgov et al., 2017). Their uptake and degradation by MSCs led to the induction of the cytoprotective enzyme heme oxygenase-1 (HO-1) and stimulation of mitochondrial biogenesis (Mahrouf-Yorgov et al., 2017). Reactive oxygen species released by cells under oxidative stress and inflammation may also trigger mitochondrial donation (Paliwal et al., 2018). However, little is known about the intrinsic signaling mechanisms of mitochondrial transfer. It was shown that release of extracellular mitochondrial particles mediated by a calcium-dependent mechanism involving CD38 and cyclic ADP ribose signaling may play a key role (Hayakawa et al., 2016).

Based on published studies, it has been proposed that cell stress is required for organelle transfer. However, accumulating evidence suggests that mitochondrial transfer from MSCs also occurs under normal physiological conditions. Unidirectional transfer of intact mitochondria was observed from MSCs to PBMCs (Court et al., 2020), 56% in CD4+ cells, 17% in CD8+T cells, and 24% in B cells (Luz-Crawford et al., 2019), as well as to corneal endothelial cells (CECs), 661W cells (a photoreceptor cell line) and ARPE-19 cells (a retinal pigment epithelium cell line) (Jiang et al., 2020), primary astrocytes and neurons (Gao et al., 2019), and human umbilical cord vein endothelial cells (Feng et al., 2019) in coculture conditions.

Mitochondrial transfer from MSCs to recipient cells induced elevation of mitochondrial membrane potential, increased respiration, and improved energy metabolism as a result. Mitochondrial donation to the immune cells additionally led to metabolic and function alterations with acquisition of an anti-inflammatory phenotype. It is known that the mitochondrial metabolism influences stem cell fate and regulates pluripotency (Zhang et al., 2018). However, studies investigating the role of MSC-derived mitochondria on the morphology of recipient cells and their properties are few. Konari et al. (2019) observed that transfer of isolated mitochondria caused structural restoration of renal proximal tubular epithelial cells (PTECs) and the structure of the tubular basement membranes and brush borders *in vivo*. We believe that the influence of mitochondrial donation by MSCs on recipient cell morphology, physiological properties, and mitochondria-dependent metabolic reprogramming warrants further study in the future.

The ability of MSCs to transfer mitochondria may be enhanced by upregulation of Miro1 (adaptor protein participating in mitochondria moving along microtubules) (Ahmad et al., 2014), which can be primed in a number of ways including coculturing MSCs with the target cells (Babenko et al., 2015), under high level of TNFα-IP2 expression (Zhang et al., 2016), by antioxidant treatment (N-acetyl-L-cysteine and L-ascorbic acid 2-phosphate) of MSCs (Li et al., 2017), by TNF-α treatment (induce TNTs formation) (Melcher et al., 2017), or damaged somatic cell-derived mitochondria (Mahrouf-Yorgov et al., 2017). Modulation of mitochondrial donation capacity might be one route to increasing the therapeutic potential of MSCs.

In theory, even one functional mitochondrion transferred into a recipient cell may propagate, due to the evolutionarily conserved mechanism for the selective amplification of wild-type mtDNA (Hill et al., 2014). However, this assumption still needs to be experimentally verified. Recently, the fate of delivered foreign mitochondria in target cells was investigated by Jiang et al. (2020) using mitochondria Cyto-Tracer. The authors demonstrated that transferred mitochondria were either digested by the host lysosomes or expelled from the cell within 3–5-μm round bubbles after 8 days (Jiang et al., 2020). However, the main difficulty in the mitochondrial tracking studies is attenuation of fluorescent signal in recipient cells due to mitochondrial division. To detect the mitochondrial heteroplasmy in recipient cells and evaluate the lifespan of the foreign mitochondria in the recipient cells, more sensitive methods of detection such as sequencing and isotope labeling may provide a clearer picture.

## Mechanism of Mitochondrial Transfer

**FIGURE 1:**
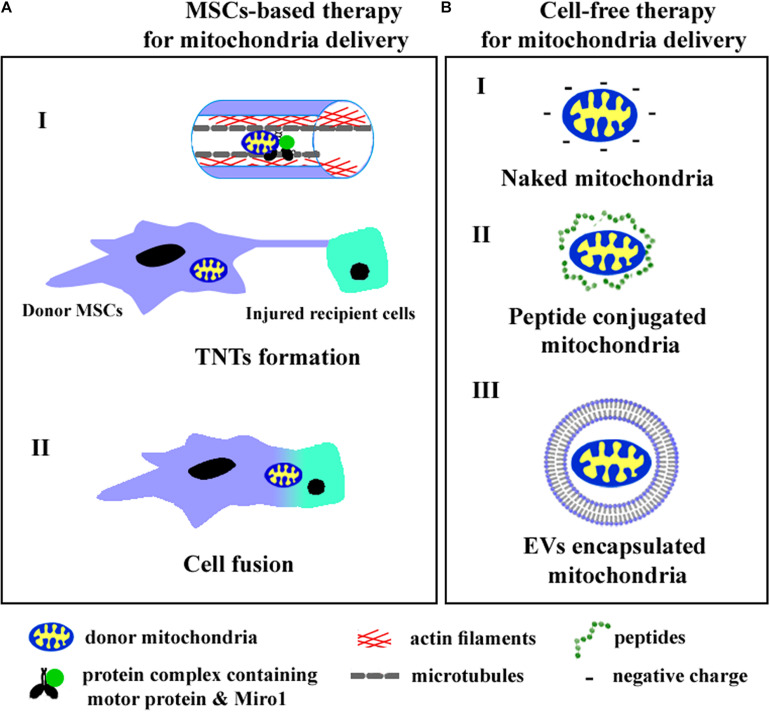
The cell-based **(A)** and cell-free **(B)** strategies of mitochondria delivery into recipient cells. **A (I)**—mitochondria transfer through TNTs, **A (II)**—mitochondria exchange after cell fusion, **B (I)**—injection of isolated mitochondria, **B (II)**—application of peptide conjugated mitochondria, **B (III)**—delivery of mitochondria encapsulated into EVs.

Intercellular mitochondrial trafficking occurs via tunneling nanotubes (TNTs), extracellular vesicles (EVs), and cellular fusion. Recently, cell and cytoplasmic membrane-free respiratory competent mitochondria were observed in blood and conditioned cell culture medium (Al Amir Dache et al., 2020). Although the role of cell-free mitochondria in intercellular communication remains to be fully understood, the practical approaches aimed to transfer intact mitochondria into target cells have been previously developed. We summarize the known ways of mitochondrial transfer into recipient cells in Figure 1.

### Mitochondrial Transfer via TNTs

TNTs are intercellular, actin, or microtubule-based cytoplasmic channels enveloped by a cytoplasmic membrane, connecting cells and forming intercellular transport networks (Vignais et al., 2017; Jash et al., 2018). TNTs can be formed by thin filaments of F-actin and a thicker subset (0.7 μm) of both F-actin and microtubules (Onfelt et al., 2004; Wang et al., 2018). The first type are called actin-based TNTs (AC-TNTs); the last type are microtubules containing TNTs (MT-TNTs) (Rustom, 2016). AC-TNTs are characterized by a limited lifespan and transfer of small molecules, organelles, and ions, whereas MT-TNTs have an increased diameter, have a prolonged lifespan, and transfer larger organelles such as mitochondria (Rustom, 2016). MT-TNTs were first described by Wang et al. in the context of a long-distance transport of mitochondria from control cells to rescue of apoptotic pheochromocytoma (PC12) cells, stressed by UV radiation (Wang and Gerdes, 2015; Rustom, 2016). Since then, numerous investigations have shown mitochondrial donation via TNTs, these are summarized in Table 1.

**TABLE 1:** Studies demonstrating TNT-mediated mitochondria transfer.

Donor cell	Recipient cell	Conditions	References
Human endothelial progenitor cells	Rat cardiomyoctes	Normal conditions, *in vitro*	Koyanagi et al., 2005
Human mesenchymal stem cells (hMSCs)	Rat cardiomyocytes	Normal conditions, *in vitro*	Plotnikov et al., 2008
Mesenchymal multipotent stromal cells	Renal tubular cells	Normal conditions, *in vitro*	Plotnikov et al., 2010
Mouse endothelial progenitor cells	Stressed human endothelial cells	(1) Exposure to glycated collagen I *in vitro* (2) Streptozotocin (STZ)-induced diabetes in mice	Yasuda et al., 2011
Mouse bone marrow-derived stromal cells (mBMSCs)	Alveolar epithelium	Sepsis model of acute lung injury *in vivo*	Islam et al., 2012
MSCs and endothelial cells	Cancer cells (MCF7, MDA, OVCAR3, SKOV3)	Normal conditions, *in vitro*	Pasquier et al., 2013
Human-induced pluripotent stem cell-derived MSCs	Bronchial epithelial cells (BEAS-2B)	(1) Injury induced by cigarette smoke medium *in vitro* (2) Model of cigarette smoke–induced lung damage *in vivo*	Li et al., 2014
MSCs	Bronchial epithelial cells	(1) Mitochondrial dysfunction induced by pro-inflammatory supernatant *in vitro* (2) Mouse model of mitochondrial dysfunction and lung injury *in vivo*	Ahmad et al., 2014
MSCs	Human umbilical vein endothelial cells	Ischemia–reperfusion model *in vitro*	Liu et al., 2014
Mesenchymal multipotent stromal cells	Rat neural cells	(1) Normal conditions, *in vitro* (2) Middle cerebral artery occlusion model of focal ischemia *in vivo*	Babenko et al., 2015
MSCs and iPSC-MSCs	Mouse cardiomyocytes	(1) Doxorubicin-induced Injury *in vitro* (2) Mouse model of anthracycline-induced cardiomyopathy *in vivo*	Zhang et al., 2016
hMSCs	Human lung epithelial cells (BEAS2B)	Normal conditions, *in vitro*	Sinclair et al., 2016
MSC	Lung macrophages	(1) Normal conditions, *in vitro* (2) Acute respiratory distress syndrome *in vivo*	Jackson et al., 2016
MSCs	Corneal epithelial cells	Rotenone-induced oxidative stress *in vitro*	Jiang et al., 2016
BM-MSCs	H9c2 cardiomyocytes	Ischemia/reperfusion injury *in vitro*	Han et al., 2016
Bone marrow stromal cells	Acute myeloid leukemia blasts	Normal conditions, *in vitro*	Marlein et al., 2017
hMSC	hMSCs	H_2_O_2_ induced oxidative stress *in vitro*	Li et al., 2017
Wharton’s jelly MSCs	Mitochondria-deficient cells (mutation in mitochondrial DNA)	Cells from patient with MERRF syndrome, *in vitro*	Chuang et al., 2017
MSCs	T-cell acute lymphoblastic leukemia cells	Normal conditions, *in vitro*	Hough et al., 2018
iPSC-MSCs	Human BEAS-2B bronchial epithelium	(1) CoCl_2_-induced mitochondrial dysfunction *in vitro* (2) Model of asthma inflammation in mice *in vivo*	Yao et al., 2018
Human iPSC-MSCs	Rat neuroendocrine PC12 cells	CoCl_2_-induced cell damage *in vitro*	Yang et al., 2020

*BM-MSCs, bone marrow-derived MSCs; AD-MSCs, adipose-derived MSCs; iPSC-MSCs, induced pluripotent stem cell-derived MSCs.*

Ahmad et al. (2014) investigated the molecular mechanisms of mitochondrial donation, demonstrating that Miro1 is essential for mitochondrial transport. The lack of Miro1 retarded mitochondrial movement through TNTs and abolished the MSC therapeutic effect. Yao et al. (2018) showed that connexin 43 regulates TNT formation, since its knockdown diminished TNT formation in human-induced pluripotent stem cells (iPSCs) and derived MSCs. Mitochondrial transfer was reduced in all cocultures after microtubule/TNT or endocytosis inhibition (Sinclair et al., 2016; Jackson and Krasnodembskaya, 2017).

### Mitochondrial Transfer via EVs

EVs are a heterogeneous group of bilipid membrane vesicles, encapsulating proteins and genetic material, as well as organelles, including mitochondria (Islam et al., 2012), ribosomes (Court et al., 2008), and proteasomes (Yu et al., 2014). EVs transfer biomolecules and organelles to target cells to mediate long-distance intercellular cross talk. Large EVs 100–1,000 nm in size (microparticles) are able to encapsulate mitochondria which are on average nearly 500 nm in size (Vignais et al., 2017). Falchi et al. (2013) also described large shedding vesicles (1–8 μm in diameter) that contained mitochondria in cultures of human fetal astrocytes.

Islam et al. (2012) first described the EV-mediated transfer of mitochondria from BMSCs to injured lung alveolar epithelial cells in a model of LPS-induced acute lung injury. The same LPS-induced acute lung injury model was also used by Morrison et al. (2017) to demonstrate the mechanism of action of MSC-derived EVs in the amelioration of lung injury. The authors showed that the transfer of mitochondria from MSCs to macrophages was mediated by EVs (Morrison et al., 2017). Hayakawa et al. (2016) also observed the presence of extracellular particles containing mitochondria in conditioned medium from rat cortical astrocytes *in vitro*. The authors also demonstrated that astrocytes in mice can release functional mitochondria that enter neurons in a mouse model of focal cerebral ischemia (Hayakawa et al., 2016). Phinney et al. (2015) showed that encapsulating of mitochondria into EVs might be a rescue mechanism from oxidative stress and clearance of depolarized mitochondria.

There have been multiple reports of EVs carrying and delivering mitochondria to a wide range of cell types. EVs of myeloid-derived regulatory cells (MDRCs) delivered mitochondria to recipient T cells *in vitro* (Hough et al., 2018). EV-mediated mitochondrial transfer was detected from renal scattered tubular cells to tubular epithelial cells *in vitro* and to stenotic kidney *in vivo* causing a protective effect, restoring mitochondrial function *in vitro*, and improving perfusion and oxygenation *in vivo* (Zou et al., 2018). Zhang et al. (2020) showed that an average 83.11% of HSC-derived EVs, B cell-derived EVs, and T cell-derived EVs carry respiring mitochondria. Neural stem cells (NSC) deliver functional mitochondria to target cells via Mito-EVs increasing Rho^0^ cell survival *in vitro* and ameliorating clinical deficits in a mouse model of autoimmune encephalomyelitis (Peruzzotti-Jametti et al., 2020). However, the mechanisms of how mitochondria are encapsulated into EVs remains insufficiently investigated.

### Cell Fusion

Acquistapace et al. (2011) showed that under coculture of mouse-differentiated cardiomyocytes with human multipotent adipose-derived stem cells, cell fusion occurs. The authors showed that as a result of mitochondrial transfer into cardiomyocytes, the resulting hybrid cells were reprogrammed to a progenitor-like state (Acquistapace et al., 2011). To date, several studies have shown that stem cells can fuse with neurons (Cusulin et al., 2012) and hepatocytes (Terada et al., 2002), forming hybrid cells which recapitulate traits specific for stem and differentiated cells (Murray and Krasnodembskaya, 2019). It was shown that length of intercellular connection is inversely proportional to the number of transferred mitochondria: elongation of the distance between cells led to fewer mitochondria being transferred (Wada et al., 2017). Cell fusion results in massive mitochondrial delivery into recipient cells. Cell fusion is a rare event under normal conditions, but hypoxia-induced apoptosis (Noubissi et al., 2015), chronic inflammation (Weimann et al., 2003), or irradiation (Alvarez-Dolado et al., 2003) markedly increased it. Increased cell fusion events between MSCs and differentiated cells as a consequence increase the mitochondria transfer and tissue restoration with heterokaryons detected in regenerated tissue.

## Therapy Based on Isolated Mitochondria

Replacement of damaged mitochondria with isolated functional mitochondria which could be internalized by targeted cells has been proposed to treat mitochondrial diseases. Autologous respiration competent mitochondria (naked mitochondria) isolated from non-ischemic tissue and injected directly into the ischemic myocardium can protect the heart from ischemia–reperfusion injury (Masuzawa et al., 2013). In neurodevelopmental diseases, systemic administration of isolated mitochondria improved the endurance of mice and prevented the progression of Parkinson disease by increasing the activity of the electron transport chain, decreasing reactive oxygen species levels, and preventing cell apoptosis and necrosis (Shi et al., 2017). Animal models of schizophrenia show that intra-prefrontal cortex injection of isolated mitochondria prevents the decrease of mitochondrial potential and attentional deficit at adulthood (Robicsek et al., 2018). The introduction of isolated mitochondria in rats with doxorubicin-mediated nephrotoxicity found that mitochondrial transplantation in the renal cortex decreased cellular oxidative stress and promoted regeneration of tubular cells (Kubat et al., 2020). Even xenogenic mitochondria restored the motor activity and mitigated the brain infarct area and neuronal cell death (Huang et al., 2016).

The first clinical application of mitochondrial autotransplantation was carried out in 2017 in Boston Children’s Hospital (United States) to treat myocardial ischemia–reperfusion injury in pediatric patients (Emani et al., 2017). Mitochondria were isolated from the patients’ non-ischemic skeletal muscle and injected directly into the injured myocardium. The authors observed improvement of ventricular function and no adverse complications (i.e., arrhythmia, intramyocardial hematoma, or scarring) (Emani et al., 2017). A second clinical trial was initiated in 2018 in Sun Yat-sen University (China) to improve oocyte quality. The procedure included the microinjection of autologous mitochondria from bone marrow MSCs into human sex cells (oocyte and sperm) (ClinicalTrials.gov Identifier: NCT03639506).

In general, naked mitochondria show low internalization ratios into target cells due to the negative surface charge. Therefore, peptide-mediated mitochondrial delivery (Chang et al., 2013), magnetic nanoparticles (Macheiner et al., 2016), and centrifugation-based (Kim et al., 2018) approaches were applied to enhance the efficiency of naked mitochondrial delivery. Peptide labeling of mitochondria was applied by Chang et al. (2016) before injection into rat brains. The authors showed significant enhancement of the survival of dopaminergic neurons and support of mitochondrial function after mitochondria were injected into a mouse model of Parkinson’s disease (Chang et al., 2016). More recently, biocompatible polymers (dextran with lipophilic cation triphenylphosphonium) have been suggested as a more effective strategy of coating of isolated mitochondria to improve uptake (Wu et al., 2018).

## Mitochondrial Delivery Strategies

Since mitochondrial donation by stem cells has been demonstrated to play a significant role in rescuing injured cells and tissues, stem cell transplantation was suggested as one of the perspective approaches for mitochondrial delivery. Joerg et al. showed that allogeneic hematopoietic stem cell transplantation restored mitochondrial function and improved clinical symptoms in patients with mitochondrial neurogastrointestinal encephalomyopathy (Halter et al., 2015). According to ClinicalTrials.gov, there were the following ongoing clinical trials of cell therapy for the treatment of mitochondrial dysfunction and mitochondria-associated diseases: Pearson syndrome (NCT03384420), ophthalmic pathology (including age-related macular degeneration, glaucoma) (NCT03011541), inherited metabolic disorders (including mitochondrial neurogastrointestinal encephalopathy) (NCT02171104)^1^. However, a major challenge of any stem cell-based therapy is oncogenic transformation, undesired differentiation, and blood vessel occlusion, which have to date limited their clinical use (Gomzikova et al., 2019).

Stability of naked mitochondria in serum was a key question for its successful therapeutic application. Shi et al. (2017) showed that incubation of naked mitochondria in serum did not significantly impair the membrane potential of mitochondria during at least 2 h of observation. Results obtained by Ishikawa et al. (2010) raised the question of mitochondrial immunogenicity. The authors demonstrated that tumor cell transplants with polymorphisms of mtDNA (mitochondria were replaced) were rejected from the host mice by the innate immune system with suppression of tumor formation. In addition, it was shown that circulating mitochondrial formyl peptides and mtDNA are recognized as damage-associated molecular patterns (DAMPs) and cause inflammatory responses identical to those activated in sepsis (Zhang et al., 2010; Wang et al., 2018).

Findings in recent years have demonstrated that EVs derived from MSCs could be the most suitable instruments for the delivery of mitochondria into damaged tissues. The membrane of EVs keeps the integrity and functional activity of the mitochondria intact, increasing their lifespan in the bloodstream. However, for the clinical development of EV-mediated mitochondrial delivery, it is necessary to overcome the challenge of obtaining sufficient quantities of EVs containing mitochondria.

MSCs are an attractive source for EV isolation due to their non- or low immunogenicity and ability to proliferate well *in vitro*. MSCs may be grown in sufficient quantities for the subsequent isolation or enrichment of EVs containing mitochondria (Wang et al., 2018). However, this is an often time-consuming and expensive approach. There are a number of approaches that can increase the enrichment of EVs containing mitochondria; these include using magnetic separation (Hubbard et al., 2019), differential centrifugation (Djafarzadeh and Jakob, 2017), or centrifugation in density gradient (Kristian, 2010) to separate those larger EVs capable of carrying intact mitochondria. There are also a number of techniques that induce the artificial production of EVs from MSCs, which are capable of carrying mitochondria and are reliably produced in much greater quantities. These approaches have been reviewed in detail previously (Gomzikova et al., 2019). Combining the approaches of increased EV isolation with enrichment would enable the creation of a therapeutic mitochondrial treatment with the potential for robust clinical application.

Mitochondrial transplantation strategies based on the systemic injection of isolated functional mitochondria, stem cells, or EVs still do not possess a specificity of delivery and will affect a variety of cells, such as blood cells and vessel-rich organs such as the lung and liver. Previously, it was shown that the intravenous administration of isolated mitochondria caused the mitochondria to become trapped in the lungs (Zhu et al., 2016), while the therapeutic efficacy of mitochondrial transplantation for the treatment of tissue injury or mitochondria-associated disorders will benefit from the targeted delivery of mitochondria into a specific tissue or organs. Due to the inherited characteristics of mitochondrial diseases and the presence of defective mitochondria in every cell of an organism, specificity of mitochondrial delivery is not always strictly necessary. In these cases, achieving sufficient systemic distribution remains a clear obstacle. We suppose that a major focus of future research will be the development of delivery strategies or vectors to target specific cells or overcome the challenges of systemic distribution.

## Conclusion

Mitochondrial transfer is a prospective strategy for the treatment of tissue injury, mitochondrial diseases, and mitochondria-associated disorders. Single healthy delivered mitochondria can cause the amplification of functional mitochondria in recipient cells and rescue the phenotype of mitochondrial deficient cells. Development of efficient mitochondrial delivery protocols is a key task for the translation of recent findings into appropriate clinical applications.

## Author Contributions

MG conceived the idea, wrote the manuscript, and created the tables and the figure. VJ edited the manuscript. AR provided financial support and final approval of manuscript. All authors contributed to the article and approved the submitted version.

## Conflict of Interest

The authors declare that the research was conducted in the absence of any commercial or financial relationships that could be construed as a potential conflict of interest.

## Footnotes

1 https://clinicaltrials.gov/
