# Supplementary material for: Mitochondria Donation by Mesenchymal Stem Cells: Current Understanding and Mitochondria Transplantation Strategies
Source: Front Cell Dev Biol. 2021 Apr 7;9:653322. doi: 10.3389/fcell.2021.653322 (PMC8058353; doi:10.3389/fcell.2021.653322)
Supplement: Supplementary file 1 [file Table_1.DOCX]

**Mitochondria donation: current understanding and mitochondria transplantation strategies**

**Supplementary Data**

Table 1. Summary of the development and most recent studies on mitochondrial transfer (2018-2020)

| Disease or injury model | Type of treatment | Source of mitochondria | Recipient cells / | Effect | Reference |
| --- | --- | --- | --- | --- | --- |
| Normal conditions in vitro, cranial critical-size bone defect model in vivo | isolated mitochondria | Mesenchymal stem cells | Bone marrow derived mesenchymal stem cells (BM-MSCs) | induced proliferation and migration, and increased osteogenesis upon osteogenic induction in vitro and bone formation in vivo | (1) |
| Normal conditions | isolated mitochondria | human umbilical cord derived-mesenchymal stem cells | MDA-MB-231 cells | increased cell proliferation, invasiveness and enhanced cisplatin-induced apoptosis | (2) |
| Doxorubicin‐mediated nephrotoxicity in rats | isolated mitochondria | Mesenchymal stem cells (MSCs) | infused into the renal cortex | decreased cellular oxidative stress and promoted regeneration of tubular cells | (3) |
| exposure of neurons to hydrogen peroxide | In vitro: co-culture | Human Marrow Stromal Cells | mouse neurons | increased neuronal survival, improved metabolism | (4) |
| CoCl2‐induced hypoxia | In vitro: co-culture | mesenchymal stem cells derived from induced pluripotent stem cells | PC12 cells | reduced apoptosis and restored ΔΨm, ameliorated mitochondrial swelling, the disappearance of cristae, and chromatin margination | (5) |
| Normal conditions, mice immunized with Complete Freund's Adjuvant | Co‐culture, isolated mitochondria, in vivo injection | Umbilical cord‐MSCs | PBMC in vitro, intraperitoneal injection in vivo | increases the expression of mRNA transcripts involved in T‐cell activation and T regulatory cell differentiation in a highly suppressive population | (6) |
| Normal conditions | Co‐culture | MSCs | corneal endothelial cells (CECs), 661W cells (a photoreceptor cell line) and ARPE-19 cells (a retinal pigment epithelium cell line) | increased aerobic capacity and upregulation of mitochondrial genes. | (7) |
| Expose to the reactive oxygen species (ROS)–inducing chemotherapy agents in vitro and in vivo on the model of acute lymphoblastic leukemia (ALL) | In vitro: co-culture | MSCs and the MSC cell line HS27 | B-precursor ALL cell lines: REH, SD1, SEM and TOM1 in vitro | prevents ALL cell apoptosis and death from exogenously administered ROS-inducing agents | (8) |
| Normal conditions and rheumatoid arthritis | In vitro: co-culture | MSCs from the bone marrow (BM) | T helper 17 (Th17) cells | oxygen consumption increase by Th17 cells and interconversion into T regulatory cells. | (9) |
| lipopolysaccharide (LPS)- induced model of depression | isolated mitochondria | Mice hippocampal lysate | in vivo intravenous injection | ameliorated LPS- induced depressive-like behaviors: decreased the immobility time of mice, attenuated the decrease in sucrose preference test, reduced neuroinflammation. | (10) |
| Normal conditions and Alexander disease (AxD)-associated mutations in GFAP gene | In vitro: co-culture | astrocytes (HA), neuronal cells SK-N-SH (SK) | astrocytes (HA), neuronal cells SK-N-SH (SK), primary mouse astrocytes and neurons | elevated mitochondrial membrane potential in the recipient cells. | (11) |
| Normal conditions | In vitro: co-culture | marrow stromal cell line | acute lymphoblastic leukemia cells | metabolic support, changes in genes related to energy metabolism and redox status | (12) |
| Normal conditions and cytarabine-induced stress | In vitro: co-culture | bone marrow MSCs | human umbilical cord vein endothelial cells | Reduced apoptosis, promoted proliferation and restored the migration ability and capillary formation | (13) |
| Hypoxia/reoxygenation stress | In vitro: co-culture | MSCs | neonatal mouse cardiomyocytes | anti-apoptosis effect | (14) |
| Middle cerebral artery occlusion (MCAO) and reperfusion model of ischemic stroke | In vivo: intra-arterial injection | MSCs | In vivo: intra-arterial injection | improved mitochondrial activity of injured microvasculature, enhanced angiogenesis, reduced infarct volume, and improved functional recovery | (15) |
| Streptozotocin (STZ)-induced diabetic animals | Isolated mitochondria (injected under the renal capsule of STZ), In vitro: co-culture, | bone marrow-derived mesenchymal stem cells (BM-MSCs) | renal proximal tubular epithelial cells | enhanced the expression of mitochondrial superoxide dismutase 2 and Bcl-2 expression and inhibited reactive oxygen species (ROS) production in vitro. mproved the cellular morphology of STZ-PTECs, and the structure of the tubular basement membrane and brush border in vivo. | (16) |
| rotenone-stressed fibroblasts of a MELAS (Mitochondrial myopathy, encephalomyopathy, lactic acidosis, and stroke-like episodes) patient | In vitro: co-culture, | Wharton's jelly mesenchymal stem cells (WJMSCs) | Fibroblasts were isolated from a MELAS patient skin punch biopsy. | mutation burden of MELAS fibroblasts was reduced to an undetectable level, with long-term retention. Improves mitochondrial functions and cellular performance, including protein translation of respiratory complexes, ROS overexpression, mitochondrial membrane potential, mitochondrial morphology and bioenergetics, cell proliferation, mitochondrion-dependent viability, and apoptotic resistance. | (17) |
| Normal conditions and starvation-induced endocytosis | isolated mitochondria | normal human astrocytes | human glioma cells (U87) | enhanced gene and protein expression related to the tricarboxylic acid (TCA) cycle, increased aerobic respiration, attenuated glycolysis, reactivated the mitochondrial apoptotic pathway, inhibited malignant proliferation. | (18) |
| Knockout and wild type mice | in vivo: intravitreal injection | induced pluripotent stem cell-derived MSC | injected into the vitreous cavity of one eye | RGC survival was significantly increased with improved retinal function. | (19) |
| Oxygen-glucose deprivation (OGD) injured neurons/ ischemic injury of the spinal cord in vivo | Isolated mitochondria, In vitro: co-culture, injection into the spinal cord in vivo | bone marrow mesenchymal stem cells (BMSCs) | OGD injured neuron, injection into the spinal cord in vivo | improved the bioenergetics profile, decreased apoptosis and promoted cell survival in post-OGD motor neurons in vitro. improved locomotor functional recovery in SCI rats in vivo. | (20) |
| Model of middle cerebral artery occlusion (MCAO) in rat | Injection into lateral ventricles in vivo | Isolated mitochondria from autologous biopsies of muscle samples derived from the pectoralis | Injection into the left lateral ventricle in vivo | reduced cellular oxidative stress and apoptosis, attenuated reactive astrogliosis, decreased brain infarct volume, reversed neurological deficits and promoted neurogenesis in vivo | (21) |
| Cisplatin damage in vitro and in vivo | intranasal administration | MSCs | neural stem cells in vitro, intranasal administration in vivo | decreases cisplatin-induced NSC death, reversed decrease in mitochondrial membrane potential in vitro. Prevented the loss of DCX+ neural progenitor cells in vivo. | (22) |
| high-fat diet of animals in order to elicit metabolic inflammation | Co-culture in vitro, Intravenous injection in vivo | MSCs | skin fibroblasts from mitochondrial disease patient in vitro, Intravenous injection in vivo | rescues impaired mitochondrial morphology, enhances host metabolic capacity, and induces widespread host gene shifting. | (23) |
| Hypoxia/reoxygenation-induced cardiomyocytes apoptosis | Co-culture in vitro | cardiac myofibroblasts | Neonatal rat cardiomyocytes | apoptosis was attenuated | (24) |
| Normal conditions | Co-culture in vitro | airway myeloid-derived regulatory cells | CD4+ T cells | transfer of mitochondria packaged within exosomes | (25) |
| Chemotherapeutic drugs caused intracellular oxidative stress | Co-culture in vitro | Bone marrow mesenchymal stem cells | Jurkat cells | Increased chemoresistance | (26) |
| Normal conditions | isolated mitochondria coated with a polymer (dextran with lipophilic cation triphenylphosphonium) | HeLa cells, liver and skeletal muscle | MDA‐MB‐231, SUM‐159PT, H9c2 rat heart myoblast cells, L929 mouse fibroblast cells adult mouse CMs | protects the organelles and facilitates cellular internalization compared with uncoated mitochondria. improved oxidative phosphorylation, and reduced glycolysis. | (27) |
| Model of schizophrenia | isolated mitochondria | Lymphoblasts, rat brain (except the cerebellum) | intra-prefrontal cortex injection in vivo | prevents decrease of mitochondrial potential and attentional deficit at adulthood | (28) |
| Antimycin-A (AMA)-induced injury in vitro; unilateral renal artery stenosis in vivo | Co-culture in vitro; intra-arterially injection in vivo | renal scattered tubular cells (STC-like cells) | tubular epithelial cells (PK1 cells) in vitro; intra-arterially injection in vivo | viability, ATP production were improved, attenuated oxidative stress  improved perfusion and oxygenation of stenotic kidney in vivo | (29) |

**References:**

1. Guo, Y., et al., *Mitochondria transfer enhances proliferation, migration, and osteogenic differentiation of bone marrow mesenchymal stem cell and promotes bone defect healing.* Stem Cell Res Ther, 2020. **11**(1): p. 245.

2. Kheirandish-Rostami, M., et al., *Mitochondrial characteristics contribute to proliferation and migration potency of MDA-MB-231 cancer cells and their response to cisplatin treatment.* Life Sci, 2020. **244**: p. 117339.

3. Kubat, G.B., et al., *The effects of mesenchymal stem cell mitochondrial transplantation on doxorubicin-mediated nephrotoxicity in rats.* J Biochem Mol Toxicol, 2020: p. e22612.

4. Tseng, N., et al., *Mitochondrial transfer from mesenchymal stem cells improves neuronal metabolism after oxidant injury in vitro: The role of Miro1.* J Cereb Blood Flow Metab, 2020: p. 271678X20928147.

5. Yang, Y., et al., *Transfer of mitochondria from mesenchymal stem cells derived from induced pluripotent stem cells attenuates hypoxia-ischemia-induced mitochondrial dysfunction in PC12 cells.* Neural Regen Res, 2020. **15**(3): p. 464-472.

6. Court, A.C., et al., *Mitochondrial transfer from MSCs to T cells induces Treg differentiation and restricts inflammatory response.* EMBO Rep, 2020. **21**(2): p. e48052.

7. Jiang, D., et al., *Bioenergetic Crosstalk between Mesenchymal Stem Cells and various Ocular Cells through the intercellular trafficking of Mitochondria.* Theranostics, 2020. **10**(16): p. 7260-7272.

8. Burt, R., et al., *Activated stromal cells transfer mitochondria to rescue acute lymphoblastic leukemia cells from oxidative stress.* Blood, 2019. **134**(17): p. 1415-1429.

9. Luz-Crawford, P., et al., *Mesenchymal stem cell repression of Th17 cells is triggered by mitochondrial transfer.* Stem Cell Res Ther, 2019. **10**(1): p. 232.

10. Wang, Y., et al., *Mitochondrial transplantation attenuates lipopolysaccharide- induced depression-like behaviors.* Prog Neuropsychopharmacol Biol Psychiatry, 2019. **93**: p. 240-249.

11. Gao, L., et al., *Mitochondria Are Dynamically Transferring Between Human Neural Cells and Alexander Disease-Associated GFAP Mutations Impair the Astrocytic Transfer.* Front Cell Neurosci, 2019. **13**: p. 316.

12. Usmani, S., et al., *Support of acute lymphoblastic leukemia cells by nonmalignant bone marrow stromal cells.* Oncol Lett, 2019. **17**(6): p. 5039-5049.

13. Feng, Y., et al., *Human Bone Marrow Mesenchymal Stem Cells Rescue Endothelial Cells Experiencing Chemotherapy Stress by Mitochondrial Transfer Via Tunneling Nanotubes.* Stem Cells Dev, 2019. **28**(10): p. 674-682.

14. Zhang, J., et al., *Differential roles of microtubules in the two formation stages of membrane nanotubes between human mesenchymal stem cells and neonatal mouse cardiomyocytes.* Biochem Biophys Res Commun, 2019. **512**(3): p. 441-447.

15. Liu, K., et al., *Mesenchymal stem cells transfer mitochondria into cerebral microvasculature and promote recovery from ischemic stroke.* Microvasc Res, 2019. **123**: p. 74-80.

16. Konari, N., et al., *Mitochondria transfer from mesenchymal stem cells structurally and functionally repairs renal proximal tubular epithelial cells in diabetic nephropathy in vivo.* Sci Rep, 2019. **9**(1): p. 5184.

17. Lin, T.K., et al., *Mitochondrial Transfer of Wharton's Jelly Mesenchymal Stem Cells Eliminates Mutation Burden and Rescues Mitochondrial Bioenergetics in Rotenone-Stressed MELAS Fibroblasts.* Oxid Med Cell Longev, 2019. **2019**: p. 9537504.

18. Sun, C., et al., *Endocytosis-mediated mitochondrial transplantation: Transferring normal human astrocytic mitochondria into glioma cells rescues aerobic respiration and enhances radiosensitivity.* Theranostics, 2019. **9**(12): p. 3595-3607.

19. Jiang, D., et al., *Donation of mitochondria by iPSC-derived mesenchymal stem cells protects retinal ganglion cells against mitochondrial complex I defect-induced degeneration.* Theranostics, 2019. **9**(8): p. 2395-2410.

20. Li, H., et al., *Mitochondrial Transfer from Bone Marrow Mesenchymal Stem Cells to Motor Neurons in Spinal Cord Injury Rats via Gap Junction.* Theranostics, 2019. **9**(7): p. 2017-2035.

21. Zhang, Z., et al., *Muscle-derived autologous mitochondrial transplantation: A novel strategy for treating cerebral ischemic injury.* Behav Brain Res, 2019. **356**: p. 322-331.

22. Boukelmoune, N., et al., *Mitochondrial transfer from mesenchymal stem cells to neural stem cells protects against the neurotoxic effects of cisplatin.* Acta Neuropathol Commun, 2018. **6**(1): p. 139.

23. Newell, C., et al., *Mesenchymal Stem Cells Shift Mitochondrial Dynamics and Enhance Oxidative Phosphorylation in Recipient Cells.* Front Physiol, 2018. **9**: p. 1572.

24. Shen, J., et al., *Mitochondria are transported along microtubules in membrane nanotubes to rescue distressed cardiomyocytes from apoptosis.* Cell Death Dis, 2018. **9**(2): p. 81.

25. Hough, K.P., et al., *Exosomal transfer of mitochondria from airway myeloid-derived regulatory cells to T cells.* Redox Biol, 2018. **18**: p. 54-64.

26. Wang, J., et al., *Cell adhesion-mediated mitochondria transfer contributes to mesenchymal stem cell-induced chemoresistance on T cell acute lymphoblastic leukemia cells.* J Hematol Oncol, 2018. **11**(1): p. 11.

27. Wu, S., et al., *Polymer Functionalization of Isolated Mitochondria for Cellular Transplantation and Metabolic Phenotype Alteration.* Adv Sci (Weinh), 2018. **5**(3): p. 1700530.

28. Robicsek, O., et al., *Isolated Mitochondria Transfer Improves Neuronal Differentiation of Schizophrenia-Derived Induced Pluripotent Stem Cells and Rescues Deficits in a Rat Model of the Disorder.* Schizophr Bull, 2018. **44**(2): p. 432-442.

29. Zou, X., et al., *Renal scattered tubular-like cells confer protective effects in the stenotic murine kidney mediated by release of extracellular vesicles.* Sci Rep, 2018. **8**(1): p. 1263.
